# Integrated genome-wide association studies, meta-analysis, and Bayesian fine mapping reveal novel quantitative trait loci’s and functional candidate genes for vulva traits in large white pigs

**DOI:** 10.1093/jas/skaf286

**Published:** 2025-10-14

**Authors:** Jin Zhou, Xiaowen Qian, Zijian Qiu, Liming Xu, Qian Liu, Yanzhen Yin, Jinfeng Ma, Jianghui Yu, David S Casey, Lijing Zhong, Qingbo Zhao, Ruihua Huang, Pinghua Li

**Affiliations:** Key Laboratory in Nanjing for Evaluation and Utilization of Pigs Resources, Ministry of Agriculture and Rural Areas of China, Institute of Swine Science, Nanjing Agricultural University, Nanjing 210095, China; Key Laboratory in Nanjing for Evaluation and Utilization of Pigs Resources, Ministry of Agriculture and Rural Areas of China, Institute of Swine Science, Nanjing Agricultural University, Nanjing 210095, China; Key Laboratory in Nanjing for Evaluation and Utilization of Pigs Resources, Ministry of Agriculture and Rural Areas of China, Institute of Swine Science, Nanjing Agricultural University, Nanjing 210095, China; Key Laboratory in Nanjing for Evaluation and Utilization of Pigs Resources, Ministry of Agriculture and Rural Areas of China, Institute of Swine Science, Nanjing Agricultural University, Nanjing 210095, China; Key Laboratory in Nanjing for Evaluation and Utilization of Pigs Resources, Ministry of Agriculture and Rural Areas of China, Institute of Swine Science, Nanjing Agricultural University, Nanjing 210095, China; Key Laboratory in Nanjing for Evaluation and Utilization of Pigs Resources, Ministry of Agriculture and Rural Areas of China, Institute of Swine Science, Nanjing Agricultural University, Nanjing 210095, China; Key Laboratory in Nanjing for Evaluation and Utilization of Pigs Resources, Ministry of Agriculture and Rural Areas of China, Institute of Swine Science, Nanjing Agricultural University, Nanjing 210095, China; Key Laboratory in Nanjing for Evaluation and Utilization of Pigs Resources, Ministry of Agriculture and Rural Areas of China, Institute of Swine Science, Nanjing Agricultural University, Nanjing 210095, China; PIC China, Shanghai 201107, China; Jiangsu Lihua Food Group Co.,Ltd, Changzhou 213000, China; Key Laboratory in Nanjing for Evaluation and Utilization of Pigs Resources, Ministry of Agriculture and Rural Areas of China, Institute of Swine Science, Nanjing Agricultural University, Nanjing 210095, China; Key Laboratory in Nanjing for Evaluation and Utilization of Pigs Resources, Ministry of Agriculture and Rural Areas of China, Institute of Swine Science, Nanjing Agricultural University, Nanjing 210095, China; Huaian Academy, Nanjing Agricultural University, Huaian 223005, China; Key Laboratory in Nanjing for Evaluation and Utilization of Pigs Resources, Ministry of Agriculture and Rural Areas of China, Institute of Swine Science, Nanjing Agricultural University, Nanjing 210095, China; Huaian Academy, Nanjing Agricultural University, Huaian 223005, China

**Keywords:** vulva traits, candidate genes, pig, genome-wide association study, meta-analysis

## Abstract

The size and angle of the vulva are economically important traits in pig production. Gilts with small or upward-tilted vulva are typically culled directly. Selective breeding aimed at improving vulva traits can enhance the retention rate of replacement gilts. This study aimed to systematically explore the key quantitative trait loci (QTL) and genes influencing vulva traits in Large White pigs using genome-wide association studies (GWAS) and meta-analysis techniques. Data on vulva length (VL), vulva width (VW), and vulva angle scores (VAS) were collected from 2,197 Large White gilts across three distinct populations (313 from PIC, 1,169 from Topigs, and 715 from Canada), with genotyping performed using a 50K single-nucleotide polymorphism (SNP) array. The SNP-chip data were imputed to the whole-genome sequencing (iWGS) level. This study used iWGS data to conduct GWAS, identifying a genomic region (SSC5: 103.04-103.34 Mb) significantly associated with VAS in both the Topigs and Canadian Large White pig populations. The significance of this region was further strengthened through multi-population meta-analysis. The most significant SNP (rs3470833446), identified on chromosome 14 and associated with VW in PIC Large White pigs, explained 16.98% of the phenotypic variation (PVE). Multi-population meta-analysis identified novel significant SNPs associated with VL on SSC4, VW on SSC1, SSC4, and SSC6, and VAS on SSC2 and SSC5. Furthermore, a significant potential pleiotropic QTL (SSC4: 36.42-41.24 Mb) regulating both VL and VW was identified. Bayes fine mapping was employed to determine the confidence intervals for these novel QTLs, with the most refined confidence interval narrowed down to 30 kb (SSC4: 38.73-38.76 Mb for VW, and SSC5: 103.20-103.23 Mb for VAS). Based on the biological functions of the genes, the following were identified as novel regulatory candidate genes for vulva traits: *VIP*, *NAV3*, and *ESR1*. These findings reveal potential key genes and genetic mechanisms influencing vulva traits in pigs, providing a crucial molecular genetic basis for improving pig breeding and reproductive performance.

## Teaser

By identifying novel and pleiotropic QTLs and functional candidate genes associated with vulva size and angle in Large White pigs, this study enhances the understanding of the genetic mechanisms underlying these economically significant traits. The identified QTLs and genes can be leveraged in marker-assisted selection (MAS) and genomic selection (GS) to accelerate genetic advancement in vulva traits among different pig populations.

## Introduction

Pigs are vital to agricultural production, with their reproductive performance directly impacting the development and economic returns of animal husbandry. During pig reproduction, vulva traits, including length (VL), width (VW), and angle score (VAS), are critical reproductive features; gilts with small or upward-tilted vulva are typically culled directly. Graves et al. (2020) reported a significant negative correlation between pre-estrus VW and age at first estrus in gilts (*r* = −0.28, *P* = 0.01). Their research demonstrated that vulva size scores can serve as indicators of ovarian development and the onset of puberty in gilts aged 95 to 115 d. Conversely, Romoser et al. (2020) suggested that gilts with larger vulva had a higher rate of natural delivery during their first farrowing compared to those with smaller vulva (84.4% vs. 64.7%, *P* = 0.02). Additionally, gilts with larger vulva had significantly larger total litter sizes compared to those with smaller vulva (12.4 vs. 11.8, *P* = 0.02). Therefore, an in-depth investigation of pig vulva traits is of paramount theoretical and practical significance for optimizing pig production performance and reproductive efficiency.

The genetic architecture underlying most reproductive traits is intricate, thereby hindering enhancement through conventional breeding approaches. Over the past few decades, identifying single-nucleotide polymorphisms (SNP)s linked to reproductive traits and utilizing these SNPs for marker-assisted selection (MAS) or genomic selection (GS) has emerged as an effective approach in pig breeding (Suwannasing et al., 2018). However, previous studies aimed at detecting causative loci have primarily relied on low-density molecular markers to identify quantitative trait loci (QTLs), resulting in candidate loci that typically span large genomic regions with low confidence. In recent years, genome-wide association studies (GWAS) employing high-density SNP markers have been extensively applied to uncover various traits, particularly gene mutations and QTLs associated with low-heritability reproductive traits.

Research on pig vulva traits has been relatively limited to date. Corredor et al. (2020) identified multiple QTL significantly associated with vulva size on SSC2, 7, 8, and 10 in their study of Landrace replacement sow populations. Similarly, in a study on Large White replacement sow populations, they detected QTL significantly associated with vulva size on SSC1 and 5. These regions can explain at least 3.4% of the genetic variation. Yin et al. (2022) further investigated the Su-Huai pig population. They identified 9 SNPs affecting VL and VW on SSC2, 7, 9, and 13 using SNP chip data. Additionally, they discovered 11 novel QTL affecting VL and VW on SSC1, 2, 7, 8, 9, 11, 13, 16, and 17 through imputing data.

Although some candidate gene regions have been identified, our understanding of the key genes and genetic mechanisms underlying pig vulva traits remains limited. To address this gap, this study aims to systematically explore the key QTLs and genes associated with pig vulva traits using GWAS and meta-analysis. By selecting three Large White pig strains with significant market presence, we aim to identify common or large-effect loci across different strains, elucidate the genetic basis of pig vulva traits, and provide a crucial molecular genetic foundation for pig breeding and reproductive performance improvement. Ultimately, this research seeks to provide scientific support for enhancing pig production performance and the retention rate of replacement gilts.

## Materials and Methods

### Ethical approval and consent to participate

All animal experiments were performed in accordance with the Guidelines for the Care and Use of Laboratory Animals prepared by the Institutional Animal Welfare and Ethics Committee of Nanjing Agricultural University, Nanjing, China [certification no: SYXK (Su) 2022-0031].

### Animal population and data collection

**Figure 1.**
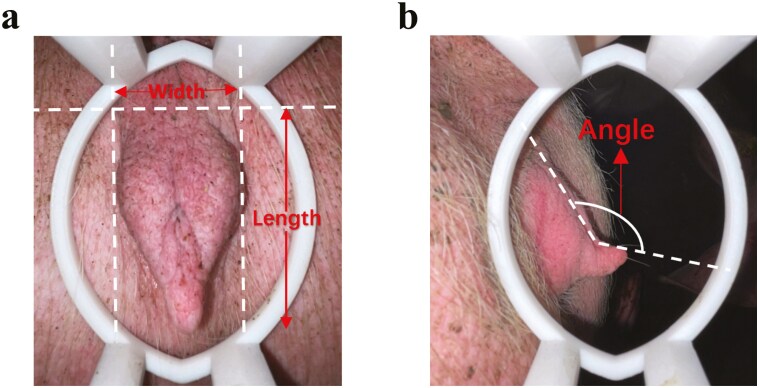
External structure of the pig vulva. Vulva length (VL) was defined as the distance from the top to the bottom of the vulva, and Vulva width (VW) was defined as the distance from the leftmost to the rightmost part of the widest portion of the vulva (A). Vulva angle (VA) was defined as the angle between the line formed by the bottom of the vulva and the bottom of the vulva cleft, and the vulva cleft (B).

Animals of the current study are the same of our previous study (Zhou et al., 2025). This study collected phenotypic data on vulva traits from a total of 2,197 gilts, including 313 PIC Large White pigs (average age: 157.07 ± 0.18 d; VAS not recorded), 1,169 Topigs Large White pigs (245.7 ± 0.62 d), and 715 Canadian Large White pigs (188.64 ± 0.32 d), with measurements taken by trained personnel. VL and VW were measured using a steel ruler with a precision of 1 millimeter. VL was defined as the distance from the top to the bottom of the vulva, and VW as the distance from the leftmost to the rightmost points of the widest portion (Figure 1). The measurement methods for VL and VW were consistent with those described by Mills et al. (2020). VAS was defined as the angle between the line formed by the bottom of the vulva and the bottom of the vulva cleft, scored through visual observation (Zhou et al., 2025). Data from gilts exhibiting estrus symptoms were excluded from the analysis. Approximately 150 milligrams of ear tissue were collected from each pig and stored in centrifuge tubes containing 75% ethanol. The PIC Large White pigs were sourced from PIC (Shanghai) Agricultural Technology Co., Ltd., the Topigs Large White pigs from Changzhou Lihua Livestock and Poultry Co., Ltd., and the Canadian Large White pigs from Jiangsu Huaizhou Wen’s Livestock Co., Ltd.

### SNP array genotyping and quality control

The genomic DNA was extracted from ear tissue samples utilizing the Megi Universal Nucleic Acid Extraction Kit. Subsequent quantification and qualification of DNA were conducted utilizing a NanoDrop 2000, adhering to standard protocols with an optimal OD260/280 ratio falling within the range of 1.7 to 2.1, and a concentration surpassing 50 ng/μl. PIC Large White pigs were genotyped using the GeneSeek GGP Porcine 50K SNP chip (Neogen), while Topigs and Canadian Large White pigs were genotyped using the Compass Porcine 50K Plus breeding chip (Tianjin, China). The physical positions of all SNPs were aligned with the Sus scrofa 11.1 build (*Sscrofa*11.1) of the pig reference genome. Quality control measures were implemented using PLINK v1.9 software (Chang et al., 2015). Individuals exhibiting SNP call rates below 90% were excluded from the analysis. Additionally, SNPs with genotype-missing rates exceeding 0.1 and minor allele frequencies below 0.05 were eliminated, retaining solely SNPs located on autosomal chromosomes. After quality control, 313 PIC individuals with 39,772 eligible SNPs, 1,169 Topigs individuals with 36,582 eligible SNPs, and 715 Canadian individuals with 38,168 eligible SNPs were used for further analysis.

### Genetic parameters estimation

This study utilized the AIREML algorithm in DMU software (Madsen, 2006) to estimate variance components, heritability, genetic and phenotypic correlations among vulva and litter traits. The statistical model of a single trait for estimating variance components was as follows:


$$\begin{array}{l} y=Xb+Zg+e,\;\;\;\;\;\;\;\;\;\;\;\;\;\;\;\left(model\;1\right) \end{array}$$

Where the vector ***y*** represents the phenotypic traits being analyzed. We define ***b*** as the vector of fixed effects, the sampling batch as the fixed effect with day age as a covariate. The vector ***g*** corresponds to the random genomic effects. The vector ***e*** signifies random residual effects. The incidence matrices are denoted as ***X*** and ***Z***. We assume that all random effects follow a normal distribution and are independent of one another. We assumed that ***g*** ~ *N* (0, $G\sigma_{G}^{2}$) and e ~ N (0, $I\sigma_{e}^{2}$), where ***G*** is the matrix of genomic relationships constructed based on SNP markers, ***I*** is the identity matrix, $\sigma_{G}^{2}$is the genomic variance, and $\sigma_{e}^{2}$ is the residual variance. The phenotypic variance ($\sigma_{p}^{2}$) was defined as the sum of $\sigma_{G}^{2}$ and $\sigma_{e}^{2}$. Heritability ($h^{2}$) was defined as the ratio of $\sigma_{G}^{2}$ and $\sigma_{p}^{2}$.

The statistical model of multiple traits for estimating variance components was as follows:


$$\begin{array}{l} \left[\begin{array}{l} y_{1}\;y_{2}\; \end{array}\right]=\left[\begin{array}{lll} X_{1} & 0\;0 & X_{2}\; \end{array}\right]\;\left[\begin{array}{l} b_{1}\;b_{2}\; \end{array}\right]+\left[\begin{array}{lll} Z_{1} & 0\;0 & Z_{2}\; \end{array}\right]\;\left[\begin{array}{l} g_{1}\;g_{2}\; \end{array}\right]+\left[\begin{array}{l} e_{1}\;e_{2}\; \end{array}\right],\;\;\;\;\;\;\;\;\;\left(model\;2\right) \end{array}$$

The parameters used in the multiple traits model are consistent with those defined in the single trait model. Specifically, $y_{i}$, $b_{i}$, $g_{i},\;X_{i}$,   and$Z_{i}$ retain their previously established meanings. We assumed that the genetic covariance matrix for 2 traits, denoted by $\left[\begin{array}{l} g_{1}\;g_{2}\; \end{array}\right]$, followed a multivariate normal distribution: N $\left(0,\;G⊗\left[\begin{array}{lll} \sigma_{G1}^{2} & \sigma_{G1G2}\;\sigma_{G1G2} & \sigma_{G2}^{2}\; \end{array}\right]\right)$. Similarly, the residual covariance matrix, $\left[\begin{array}{l} e_{1}\;e_{2}\; \end{array}\right]$, also followed a multivariate normal distribution: N $\left(0,\;I⊗\left[\begin{array}{lll} \sigma_{e1}^{2} & \sigma_{e1e2}\;\sigma_{e1e2} & \sigma_{e2}^{2}\; \end{array}\right]\right)$, $\sigma_{G1G2}$ and $\sigma_{e1e2}$ is the genomic covariance and residual covariance of the two traits, respectively. The rest of the parameters (***G***, ***I***, $\sigma_{Gi}^{2}$, $\sigma_{ei}^{2}$) have the same meanings as single trait model. Where i represents one of the traits. We defined the genetic correlation ($r_{G}$) between traits as follows:


$$\begin{array}{l} r_{G}=\frac{\sigma_{G1G2}}{\sqrt{\sigma_{G1}^{2}\times \sigma_{G2}^{2}}},\;\;\;\;\;\;\;\;\left(model\;3\right) \end{array}$$

Furthermore, the phenotypic correlation ($r_{P}$) between traits was defined as:


$$\begin{array}{l} r_{P}=\frac{\sigma_{G1G2}+\sigma_{e1e2}}{\sqrt{\sigma_{G1}^{2}+\sigma_{e1}^{2}}\times \sqrt{\sigma_{G2}^{2}+\sigma_{e2}^{2}}},\;\;\;\;\;\left(model\;4\right) \end{array}$$

### Imputation

The whole-genome resequencing data of 1,662 pigs were used as the reference panel, which includes the resequencing data of 1,602 pigs in multiple breeds from the PigGTEX project (Teng et al., 2024) and 60 Suhuai pigs (Liu et al., 2023). Haplotype phasing of reference panel (Browning and Browning 2007) and the imputation of the SNP-chip data to the whole-genome density (Browning et al. 2018) were performed using the Beagle software (version 5.2) with the default parameters. SNPs with dosage R-squared (DR^2^) < 0.9 and minor allele frequencies < 0.05 were removed. After quality control, 313 PIC individuals with 6,451,862 SNPs, 1,169 Topigs individuals with 5,710,884 SNPs, and 715 Canadian individuals with 5,797,341 SNPs were retained for further analysis.

### Genome-wide association analysis

Prior to the genome-wide association analysis (GWAS), principal component analysis was performed separately for the three populations using the genotype data from the SNP chip. This analysis was conducted using PLINK (Version 1.9) software (Purcell et al., 2007; Chang et al., 2015) to examine population structure. For experimental populations exhibiting population stratification, the first five principal components were included as covariates in the subsequent GWAS model. The LDAK (version 5.2) software (Speed et al., 2012) was used to assess the association between SNPs and phenotypes. Firstly, the SNP data were processed using the “ldak-gen” command with the “-thin” parameter to generate core SNP markers. These core SNP markers are typically SNP sites with high information content and low correlation, which are used for subsequent association analyses. Then, the ‘ldak-lassosum’ command in the LDAK software was utilized to perform association analysis based on a linear model, employing the LOCO (Leave One Chromosome Out) method. This approach avoids joint analysis of SNPs on the same chromosome, effectively balancing computational power (Yang et al., 2014) and reducing the loss of statistical power (Listgarten et al., 2012). The mixed linear model is as follows:


$$\begin{array}{l} y=Xb+Kj+g+e,\;\;\;\;\;\left(model\;5\right) \end{array}$$

Where the vector ***y*** represents the phenotypic traits being analyzed. ***b*** is a vector of fixed effects, considering age at measurement as a covariate and measurement batch as a fixed effect; the first five principal components were selected as covariates for correction. ***j*** represents the substitution effect of the alleles, ***g*** is the random polygenic effect following $g∼N\left(0,\;G\sigma_{G}^{2}\right)$ distribution, ***G*** is a kinship matrix constructed based on SNP information. ***e*** is the random residual effects following $e∼N\left(0,I\sigma_{e}^{2}\right)$ distribution, ***I*** is the identity matrix, and ***X*** and ***K*** are the incidence matrices.

The R package CMplot was used to draw Manhattan plots and Q-Q plots (Qiu et al., 2024). The genome-wide significance threshold was set using the Bonferroni correction method, defined as 0.05/N, with a suggested significance threshold set at 1/N. For chip data, N represents the number of SNPs after quality control. It is important to note that because many SNPs are in high linkage disequilibrium (LD) after imputation, the threshold obtained using the Bonferroni correction method by dividing 0.05 by the total number of detected SNPs is too stringent. Therefore, for imputed data, N, representing the number of independent SNPs, was calculated using the “--indep-pairwise 50 10 0.5’ parameter in PLINK software (Version 1.9) (Wang and Zhang, 2021), with specific values of 222,053 for the PIC population, 205,642 for the Topigs population, and 221,987 for the Canadian Large White population. The proportion of phenotypic variance explained (PVE) by SNP additive effects is calculated using the following formula:


$$\begin{array}{l} PVE=\frac{2\alpha \left(1-\alpha \right)j^{2}}{\sigma_{p}^{2}},\;\;\;\;\;\;\;\left(model\;6\right) \end{array}$$

Where α is the minor allele frequency of the SNP, ***j*** is the effect size of the SNP, and $\sigma_{p}^{2}$ is the phenotypic variance.

### Meta-analysis

Meta-analysis was conducted using METAL software to integrate GWAS information from individual population-filled datasets(Willer et al., 2010). The effect direction and *p*-value for each SNP were converted into Z-score statistics. In this process, the Z-scores of each SNP were weighted and summarized according to the sample size of each individual population. The formula for calculating the Z-score is as follows:


$$\begin{array}{l} Z_{j}=\frac{\underset{i}{\sum }\phi^{-1}\left(\frac{P_{ij}}{2}\right)\times sign\left(\Delta_{ij}\right)\times \sqrt{N_{i}}}{\sqrt{\underset{i}{\sum }N_{i}}},\;\;\;\;\;\;\;\;\left(model\;7\right) \end{array}$$

In the formula, ***i*** represents the ***i***-th population, ***j*** represents the ***j***-th SNP locus, $\Delta_{ij}$ represents the effect value of the ***j***-th SNP in the ***i***-th population, $N_{i}$ represents the population size of the ***i***-th population, ϕ represents the standard normal distribution, and $P_{ij}$ represents the *P* value of the ***j***-th SNP in the ***i***-th population. In the meta-analysis, the significance threshold was set using the strictest GWAS data from the three populations: PIC Large White population (*N* = 222,053).

### Bayes fine mapping

The Bayesian CAVIARBF method (Chen et al., 2015) was employed to identify key variant intervals associated with vulva traits. This method utilizes meta-analysis results based on imputed data from multiple populations to select a candidate variant set from SNPs significantly (*P* < 0.01) associated with traits within 1 Mb upstream and downstream of the most significant SNP in the genomic region. The CAVIARBF (Version 0.2.1) software infers the minimal set of variants containing causal mutations with 95% posterior probability using the marginal association Z-scores of the candidate variant set and LD information between SNPs. This process more accurately determines the confidence intervals of QTLs, improving the precision of QTL mapping (van de Bunt et al., 2015). The newly identified QTLs were then validated by comparing them with known QTLs in the Pig QTL database (https://www.animalgenome.org/; Hu et al., 2013) using Bayesian fine-mapping.

### Identification of candidate genes

Through conducting an in-depth review of scientific literature using PubMed (https://pubmed.ncbi.nlm.nih.gov/) and performing a systematic search in the GeneCards online database (https://www.genecards.org/), information on the biological functions of the most promising functional candidate genes was obtained.

## Results

### Descriptive statistics and heritability estimates

**Figure 2.**
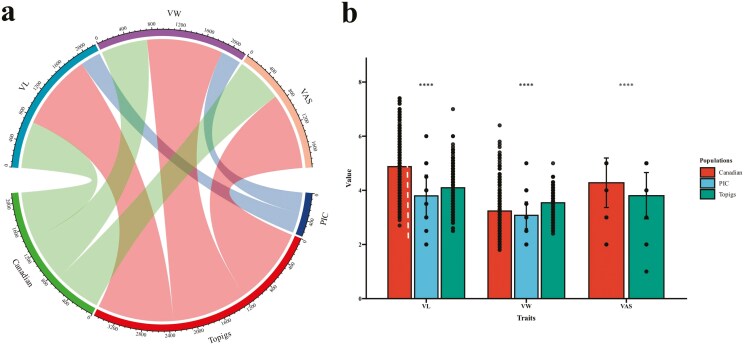
Phenotypic Statistics of Vulva Traits in Different Pig Populations. (A) Chord diagram of the phenotypic sources of vulva traits in Large White pigs. The nodes at the top of the chord diagram represent the 3 vulva traits: vulva length (VL), vulva width (VW), and vulva angle scores (VAS). The nodes at the bottom represent the 3 Large White pig populations: PIC, Topigs, and Canadian. The width of the edges connecting the upper and lower nodes indicates the number of individuals for which each trait was collected in each population. A wider edge indicates a larger number of individuals. (B) Comparison of vulva length (VL, in cm), vulva width (VW, in cm), and vulva angle scores (VAS) among PIC, Topigs, and Canadian Large White pigs.

The maximum, minimum, mean, standard error, and coefficient of variation for each parameter of VL, VW, and VAS are provided in Supplementary Table S1 and Figure 2. The heritability estimates for VL, VW, and VAS, along with their phenotypic and genetic correlation coefficients, are summarized in Table 1. The heritability of VL ranges from 0.211 to 0.302, that of VW from 0.167 to 0.301, and that of VAS from 0.297 to 0.426. VL shows significant positive correlations with both VW and VAS, with correlation coefficients exceeding 0.559 and 0.366, respectively.

**Table 1.** Genetic parameters^1^ for vulva length, vulva width, and vulva angle scores

Population	Trait	VL^2^	VW^3^	VAS^4^
PIC	VL	0.302 ± 0.118	0.767*	-
	VW	0.720*	0.167 ± 0.097	-
Topigs	VL	0.211 ± 0.041	0.585*	0.381*
	VW	0.559*	0.258 ± 0.043	−0.072
	VAS	0.376*	-0.207	0.426 ± 0.046
Canadian	VL	0.295 ± 0.059	0.670*	0.541*
	VW	0.678*	0.301 ± 0.061	0.044
	VAS	0.366*	0.145	0.297 ± 0.062

^1^Genetic correlation coefficients are listed in the upper triangle, phenotypic correlation coefficients are listed in the lower triangle, and heritability values are listed in the diagonal.* Denotes *P* < 0.05.

^2^Vulva length.

^3^Vulva width.

^4^Vulva angle scores.

### Genome-wide association studies

**Figure 3.**
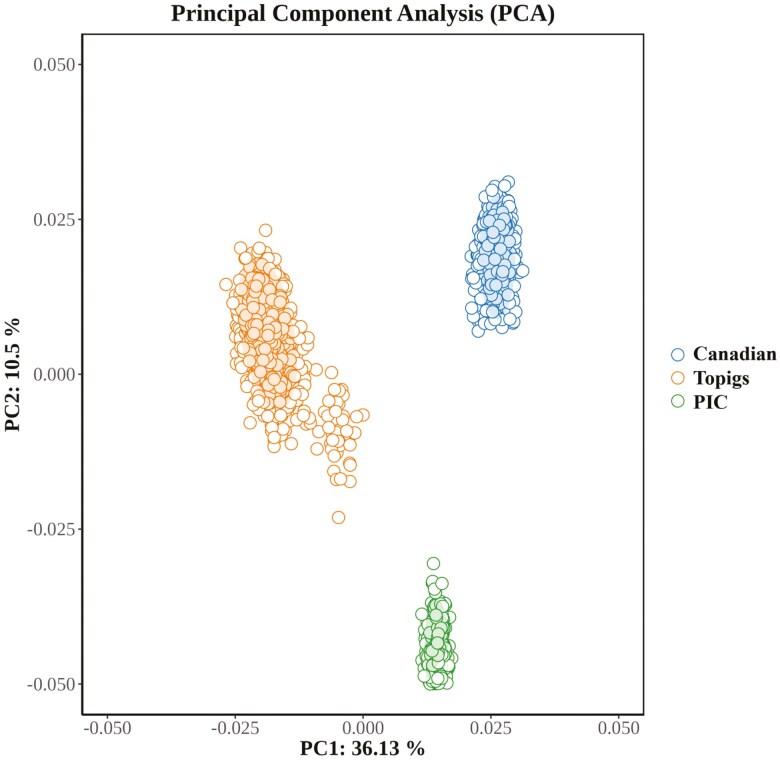
Principal component analysis (PCA) plot of population structure based on chip genotypic data, showing the top 2 principal components. PC1 and PC2 represent principal component 1 and principal component 2, respectively.

The population structure analysis of Large White pigs revealed distinct stratification among the three populations (Figure 3), indicating that they are independent populations and should be analyzed separately using GWAS. However, slight stratification was observed within the Topigs Large White pig population. Therefore, in the subsequent GWAS analysis, the first five principal components were included in the model as covariates to account for this stratification.

The average consistency rate and accuracy of genotype imputation across all populations exceeded 0.9593 and 0.9647, respectively (Supplementary Figure S1). We conducted GWAS using both chip data and iWGS data for VL, VW, and VAS. The Manhattan plots for these traits are presented in Supplementary Figures S2 and 4.

**Figure 4.**
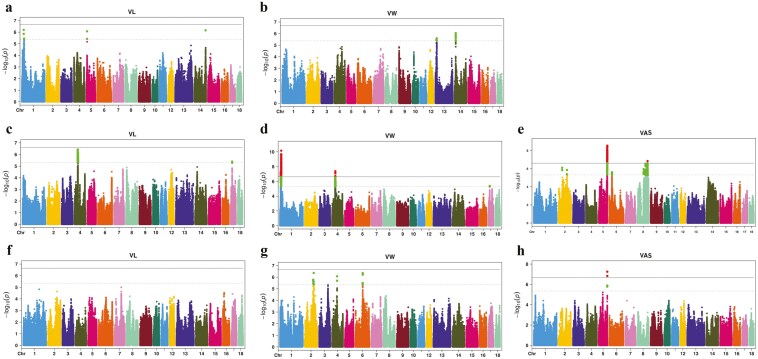
Manhattan plot of GWAS based on imputed data for vulva length (VL), vulva width (VW), and vulva angle score (VAS) traits. The results in (A) and (B) were PIC Large White pig; those in (C), (D), and (E) were Topigs Large White pig, while those in (F), (G), and (H) were Canadian Large White pig. Negative log10 *P*-values of SNPs (y-axis) were plotted against their corresponding genomic positions (x-axis). The horizontal solid and dashed lines represent the genome-wide significance and suggestive thresholds, respectively.

Based on chip data, 30 significant SNPs associated with VL were identified on chromosomes 5, 6, 8, 12, and 14 in Large White pigs from three sources, with the most significant SNP (SSC10: 102362191 bp) explaining up to 9.74% of the phenotypic variation. A total of 15 significant SNPs associated with VW were found on chromosomes 1, 2, 4, and 8. Additionally, 40 SNPs associated with VAS were located on chromosomes 1, 5, 6, 8, and 15 (Supplementary Table S2). These findings reveal previously unreported genomic regions associated with vulva traits in pigs.

Based on iWGS data, in PIC Large White pigs, 19 significant SNPs associated with VL were identified on chromosomes 5, 6, 8, 12, and 14. The most significant SNPs—rs325327092, rs344300910, and rs334365184—explained 10.53%, 9.25%, and 10.63% of the phenotypic variation, respectively. In Topigs Large White pigs, 619 significant SNPs associated with VL were identified on chromosomes 4 and 17, with the most significant SNP explaining 3.39% of the phenotypic variation. In PIC Large White pigs, 24 significant SNPs with VW were identified on chromosomes 13 and 14. The SNP rs3470833446, located within the genomic region 13.38-13.58 Mb on chromosome 14, explained 16.98% of the phenotypic variation. In Topigs Large White pigs, 1,605 significant SNPs associated with VW were identified on chromosomes 1 and 4. On chromosome 1, two genomic regions were identified at 9.02 to 9.25 Mb and 12.56-14.20 Mb. The genomic regions (SSC4: 38.76 to 38.96 Mb) were significantly associated with both VL and VW. In the Canadian Large White pig population, 117 significant SNPs associated with VW were identified on chromosomes 2 (2 genomic regions), 4, and 6, with the most significant SNPs explaining 2.12% to 2.3% of the phenotypic variation. In Topigs Large White pigs, 2,532 significant SNPs associated with VAS were identified on chromosomes 2 (two genomic regions), 5, 6, and 8 (4 genomic regions). The most significant SNP, rs324310263, was located within the genomic region 101.34 to 103.41 Mb on chromosome 5, with a *p*-value of 2.82E-09, explaining 3.6% of the phenotypic variation. In the Canadian Large White pig population, 78 significant SNPs associated with VAS were identified on chromosome 5, with rs318318582 located at 103045510 bp, explaining 13.04% of the phenotypic variation. The genomic region (SSC5: 103.04 to 103.34 Mb) associated with VAS was also identified in the Topigs Large White pig population (Table 2).

**Table 2.** GWAS results for vulva traits based on imputed data

Population	Trait	Chr^1^	Genomic region (Mb)	*N* ^2^	Top SNP	Position^3^	*P* value^4^	Var^5^
PIC	VL^6^	1	22.18 to 26.26	16	rs325327092	22333426	6.40E-07	10.53
		5	4.928 to 4.929	2	rs344300910	4929765	8.54E-07	9.25
		14	135.52	1	rs334365184	135526821	6.99E-07	10.63
	VW^7^	13	16.05 to 16.38	4	rs340749482	16316280	2.56E-06	6.83
		14	13.38 to 13.58	20	rs3470833446	13450575	9.16E-07	16.98
Topigs	VL	4	36.13 to 41.19	616	rs325032255	38949931	3.76E-07	3.39
		17	17.21 to 17.22	3	rs322862476	17219407	4.10E-06	2.63
	VW	1	9.02 to 9.25	332	rs787781987	9091205	5.02E-08	3.01
			12.56 to 14.20	747	rs692657017	14089663	6.91E-11	4.44
		4	38.76 to 38.94	526	rs343435555	38761012	4.18E-08	2.93
	VAS^8^	2	43.946 to 48.948	2	rs323371396	43948583	7.83E-07	2.51
			101.43 to 101.47	6	rs335939055	101472966	1.37E-06	1.98
		5	101.34 to 103.41	1,691	rs324310263	103415209	2.82E-09	3.6
		6	32.32 to 32.96	18	rs320249646	32327733	2.43E-06	2.27
		8	75.66 to 75.82	7	rs326031813	75668469	1.12E-06	2.08
			80.26 to 80.82	9	rs330189870	80821882	1.36E-06	2.1
			94.47 to 100.58	129	rs3475866739	98989247	2.77E-07	2.45
			111.19 to 123.23	670	rs328814984	123055001	1.42E-07	2.74
Canadian	VW	2	103.83 to 104.14	112	rs344004119	102418059	1.67E-06	2.17
			108.53 to 110.24	55	rs320192712	108546622	4.35E-07	2.25
		4	60.043 to 60.045	4	rs325361551	60043923	8.47E-07	2.3
		6	87.53 to 88.37	53	rs1110055955	88190253	4.50E-07	2.12
	VAS	5	103.04 to 103.34	78	rs318318582	103045510	5.67E-08	13.04

^1^ *Sus scrofa* chromosome, the same as below.

^2^The number of significant SNPs.

^3^Position of top SNP.

^4^ *P* value according to the Wald test.

^5^Phenotypic variation explained by the top SNP.

^6^Vulva length.

^7^Vulva width.

^8^Vulva angle scores.

### Meta-analysis

**Figure 5.**
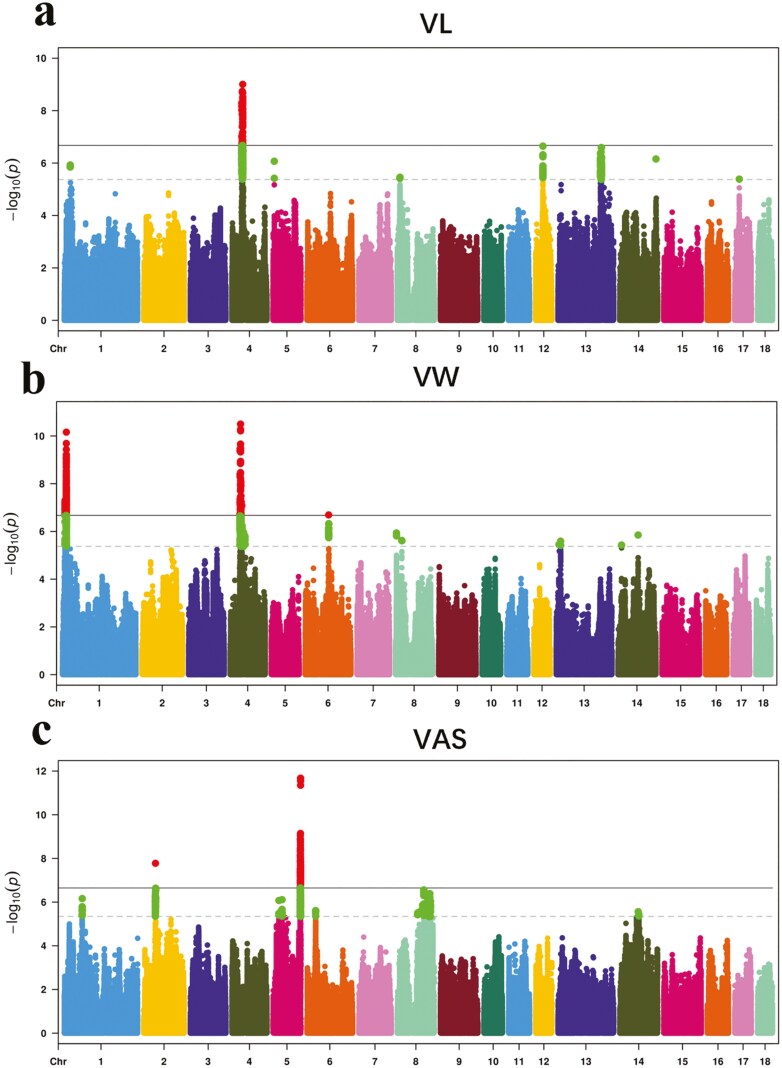
Manhattan plots of meta-analysis based on imputation data for the vulva traits. Negative log10 *P*-values of SNPs (y-axis) were plotted against their corresponding genomic positions (x-axis). The horizontal solid and dashed lines represent the genome-wide significance and suggestive thresholds, respectively.

The Manhattan plots of the meta-analysis results for VL, VW, and VAS traits, based on imputed data from different populations of Large White pigs, are shown in Figure 5. Information on significant loci exceeding the genome-wide threshold is presented in Table 3.

**Table 3.** Meta-analysis results for vulva traits based on imputed data exceeding the genome-wide significance threshold

Trait	Chr^1^	Genomic region (Mb)	*N* ^2^	Top SNP	Position^3^	*P* value^4^	Genes
VL^5^	4	36.42 to 41.24	1,541	rs318761015	40429140	9.88E-10	*CPQ, MTERF3, ENSSSCG00000049545, SDC2, GDF6, UQCRB, PTDSS1*
VW^6^	1	9.02 to 9.12	167	rs787781987	9091205	5.02E-08	*SYNJ2*
		12.69 to 14.20	704	rs692657017	14089663	6.91E-11	*MYCT1, VIP, FBXO5, ESR1, ENSSSCG00000049158, ENSSSCG00000004082*
	4	36.39 to 44.80	1,703	rs339518670	38760955	3.18E-11	*MATN2, POP1, RIDA*
	6	87.52 to 88.37	83	rs706725347	87639411	2.03E-07	*PUM1, SNRNP40, TINAGL1, SPOCD1, SERINC2, COL16A1, FABP3, PEF1, ADGRB2, ZCCHC17,HCRTR1*
VAS^7^	2	43.47 to 44.92	150	rs329773566	44036766	1.67E-08	*PSMA1, INSC, CYP2R1,COPB1,PDE3B*
	5	101.34 to 103.41	2,313	rs318823998	103201251	2.13E-12	*NAV3*

^1^ *Sus scrofa* chromosome, the same as below.

^2^The number of significant SNPs.

^3^Position of top SNP.

^4^ *P* value according to the Wald test.

^5^Vulva length.

^6^Vulva width.

^7^Vulva angle scores.

For VL, the meta-analysis of PIC, Topigs, and Canadian Large White pig populations identified 7,354 significantly associated SNPs distributed across eight chromosomes. Compared to the GWAS results from individual populations, new genomic regions were identified on chromosomes 8, 12, and 13. Additionally, the *P* value of the most significant SNP on chromosome 4 decreased to 9.88E-10. For VW, the meta-analysis identified 3,634 significantly associated SNPs distributed across 6 chromosomes and ten genomic regions. Compared to the GWAS results from individual populations, new genomic regions were identified on chromosome 8. Furthermore, the *P*-value of the most significant SNP on chromosome 4 decreased to 3.18E-11, and that of the most significant SNP on chromosome 6 decreased to 2.03E-07. However, significant loci on chromosome 2 identified in individual populations were not significant in the meta-analysis, possibly due to differences in the genetic backgrounds of the populations and the effects of different QTLs. The genomic regions (SSC4: 36.42-41.24 Mb) were significantly associated with both VL and VW. For VAS, the meta-analysis identified 2,817 significantly associated SNPs distributed across 6 chromosomes and 7 genomic regions. Compared to the GWAS results from individual populations, new genomic regions were identified on chromosome 1. Additionally, the *P*-value of the most significant SNP on chromosome 2 decreased to 1.67E-08, and that of the most significant SNP on chromosome 5 decreased to 2.13E-12.

### Bayes fine mapping

**Figure 6.**
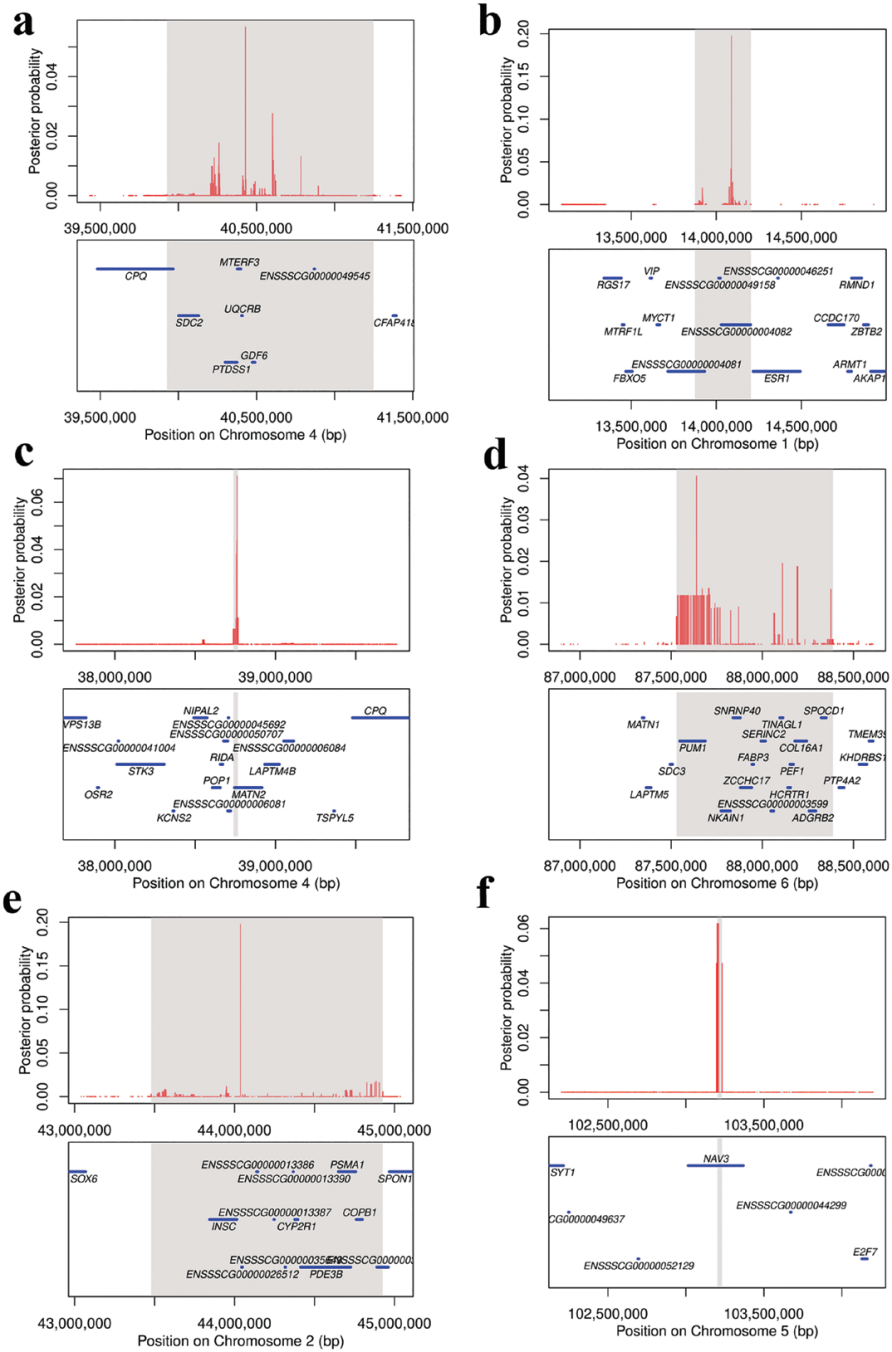
Bayes’ fine mapping of the quantitative trait loci (QTLs) regulating vulva length (A), vulva width (B, C, D), and vulva angle score (E, F). The x-axis represents the physical positions of single-nucleotide polymorphisms (SNPs) in the genome, while the y-axis indicates posterior probability. The shaded regions in each plot represent the 95% confidence intervals for the QTLs identified through Bayesian fine mapping. These intervals are the genomic regions within which the true causal variant is most likely to be located.

The results of the multi-population meta-analysis and subsequent Bayes fine mapping of QTLs significantly affecting vulva traits (exceeding the genome-wide significance threshold) are shown in Figure 6. The 95% confidence interval for the QTL associated with VL was mapped to chromosome 4, spanning 39.92 to 41.24 Mb. For VW, the QTLs were mapped to 95% confidence intervals on chromosome 1 at 13.87 to 14.20 Mb, chromosome 4 at 38.73 to 38.76 Mb, and chromosome 6 at 87.52 to 88.38 Mb. The QTLs for VAS were mapped to 95% confidence intervals on chromosome 2 at 43.47 to 44.92 Mb and chromosome 5 at 103.20 to 103.23 Mb.

### Candidate genes

According to the reported biological functions of genes in the scientific literature, *MTERF3* (Mitochondrial Transcription Termination Factor 3) and *SDC2* (Syndecan-2) were identified as potential functional candidate genes influencing VL in Large White gilts, *VIP* (Vasoactive Intestinal Peptide) and *POP1* (Processing of Precursor 1) were identified as potential functional candidate genes influencing VW, while *PSMA1* (Proteasome Subunit Alpha Type-1) was identified as a potential functional candidate gene influencing VAS.

## Discussion

### Heritability estimates

According to previous research findings, VL, VW, and VA in pigs are generally regarded as traits exhibiting moderate to high heritability (Knauer et al., 2011; Corredor et al., 2018; Yin et al., 2022). In this study, the heritability estimates for vulva traits in Large White pigs ranged from 0.167 to 0.426, aligning with the values reported in the aforementioned studies. There are differences in heritability among the different populations, potentially due to varying environmental conditions across these populations. This is particularly evident in smaller sample sizes, as environmental factors tend to have a more pronounced impact on traits, resulting in a larger proportion of environmental variance.

### GWAS on vulva Traits

Understanding the genetic mechanisms underlying complex traits in pigs is crucial for optimizing production performance, improving health and welfare, and tackling environmental challenges. Over the past decades, GWAS have emerged as indispensable tools for elucidating the genetic basis of complex traits and diseases (Bouwman et al., 2018; Wang et al., 2020a). By utilizing advanced techniques such as GWAS, we can further investigate the genetic foundations of pigs, offering both theoretical and practical insights for breeding programs (Jiang et al., 2018; Wang et al., 2018). Imputed data are valuable for identifying causal gene loci and have been extensively applied in both human and animal studies (Sanchez et al., 2017; van den Berg et al., 2019). The accuracy of imputation is affected by various factors, such as sequencing depth, reference population size, the relationship between reference and target populations, and the marker density of the target population (Deelen et al., 2014). In general, the correlation between imputed and true genotypes ranges from 0.898 to 0.952 (Pausch et al., 2017). In this study, the average concordance and accuracy across the 18 chromosomes, both before and after quality control, in three Large White pig populations, exceeded 0.9593. This suggests that the imputation quality based on chip data in this experiment is relatively high, supporting the feasibility of further genetic mutation detection using imputed data.

Compared to GWAS results derived from chip data, imputed data identified a greater number of significant SNPs. New QTLs for VL were discovered on chromosomes 1, 4, and 17, while for VW, novel QTLs were identified on chromosomes 6, 13, and 14. For VAS, a new QTL was detected on chromosome 2. Additionally, the *p*-values of certain previously significant SNPs decreased further. However, some signals that were significant in the chip data lost their significance or became less prominent in the imputed data, possibly due to the small effect of these SNPs. For most traits, associated markers typically explain only a small fraction of the phenotypic variation individually (Kutalik et al., 2011). In this study, the significant SNPs located in the QTL intervals based on imputed data could explain up to 10.63%, 16.98%, and 13.04% of the phenotypic variation for VL, VW, and VAS, respectively, which are higher than those reported in the literature related to vulva traits (Yin et al., 2022).

This study identified genomic regions that affect the same trait across multiple populations simultaneously. For example, the region SSC5: 103.04 to 103.34 Mb was significantly associated with VAS in both Topigs and Canadian Large White pigs, suggesting that this region may harbor key genes or genetic markers influencing VAS. Additionally, genomic regions influencing different traits within the same population were identified, such as a pleiotropic QTL (SSC4: 38.76 to 38.96 Mb) significantly associated with both VL and VW in Topigs Large White pigs. In pig breeding programs, selecting markers linked to these genetic variations could lead to simultaneous improvements in both VL and VW.

### Multi-population Meta-analysis

Meta-analysis maximizes the utilization of existing genomic information (Bernal Rubio et al., 2015; Le et al., 2017), enhances detection power, reduces false positive results (Blaj et al., 2018), and improves the stability and reliability of the results. A recent study conducted a comprehensive cross-breed meta-GWAS for 232 complex traits and a within-breed meta-GWAS for 12 traits, identifying 6,878 QTLs. These findings provide valuable resources and novel insights into the genetic regulation of complex traits in pigs(Xu et al., 2025). Therefore, we conducted a meta-analysis on the GWAS summary statistics derived from various sources of Large White pig populations. For VL, novel genomic regions were identified on chromosomes 8, 12, and 13, with the *P*-value of the most significant SNP on chromosome 4 decreasing to 9.88E-10. For VW, novel genomic regions were identified on chromosome 8, with the *P*-value of the most significant SNP on chromosome 4 decreasing to 3.18E-11, and that of the most significant SNP on chromosome 6 decreasing to 2.03E-07. For VAS, novel genomic regions were identified on chromosome 1, with the *P*-value of the most significant SNP on chromosome 2 decreasing to 1.67E-08, and that of the most significant SNP on chromosome 5 decreasing to 2.13E-12. The VL and VW traits in Large White pigs are influenced by the same genetic variations located within the SSC4: 36.42 to 41.24 Mb region, indicating a shared genetic regulatory mechanism. This further reflects the complexity of the genetic mechanisms underlying complex traits, where distinct traits may be influenced by the same genomic region. These findings have important implications for understanding genetic diversity in pigs and for molecular breeding efforts.

### Bayes fine mapping

One of the major challenges in identifying potential causal SNPs is the presence of LD, which can lead to highly correlated association results and multiple significant SNPs at a given locus of interest (Hutchinson et al., 2020; Zou et al., 2022). Fine-mapping methods facilitate this process by selecting and prioritizing the variants most likely to cause variation in complex traits. CAVIARBF is a Bayesian fine-mapping tool specifically designed to identify potential causal variants within associated regions (LaPierre et al., 2021). By leveraging marginal test statistics, this method can identify causal variants at multi-signal association loci. Combining traditional GWAS results with Bayesian statistical models significantly enhances the accuracy of causal variant identification. In this study, CAVIARBF was employed to refine the QTL confidence intervals of key regions identified in single-trait meta-analyses. On chromosome 4, the QTL influencing VW was narrowed to 38.73 to 38.76 Mb (30 Kb), and on chromosome 5, the QTL affecting VAS was refined to 30 Kb (103.20 to 103.23 Mb). The remaining QTLs associated with vulva traits were refined to within 1.45 Mb.

### Candidate genes

In pig production, the size and angle of the vulva are key traits, with gilts showing a small or upward-tilted vulva often culled. Selective breeding to improve vulva traits can increase the retention rate of replacement gilts. Functional candidate genes associated with vulva traits were identified by examining the functional information of all protein-coding genes within the QTL confidence intervals. *VIP* (Vasoactive Intestinal Peptide) is a multifunctional neuroendocrine peptide initially isolated from the porcine small intestine, known for its wide range of biological activities. In the reproductive system, *VIP*’s role is particularly complex and significant. *VIP* not only regulates ovarian blood flow but also modulates ovarian function by influencing neural signaling and hormone secretion (Waschek, 2013). Studies have shown that *VIP* can stimulate the cAMP/PKA signaling pathway in ovarian granulosa cells, enhancing the expression and function of the Steroidogenic Acute Regulatory protein (STAR), thereby promoting the synthesis and secretion of progesterone (P4) and estradiol (E2) (Merech et al., 2021). Additionally, *VIP* regulates the secretion of gonadotropin-releasing hormone (GnRH) and luteinizing hormone, affecting ovulation and steroidogenesis in the ovaries (Said, 2007). In patients with polycystic ovary syndrome (PCOS), *VIP* levels are significantly elevated, which may be related to the increased density of sympathetic nerve fibers in the ovaries of PCOS patients. These changes in *VIP* levels may affect normal ovarian function by modulating neural signaling and hormone levels, thereby contributing to the development of PCOS. Thus, *VIP*’s role in the ovaries extends beyond regulating hormone secretion to potentially influencing physiological and pathological processes via neural regulatory mechanisms (Sallicandro et al., 2024). *VIP* may also affect the growth and development of vulvar tissue by modulating estrogen and progesterone levels.

*NAV3* (Neuron Navigator 3) is a tumor suppressor gene widely expressed in the nervous system, primarily involved in cell migration, microtubule dynamics, and cytoskeletal anchoring (Maes et al., 2002). It exhibits abnormal expression patterns in various tumors, including gliomas, colorectal cancer, and skin cancer. *NAV3* regulates cell-matrix adhesion and cytoskeletal dynamics through its functional domains, such as calponin homology and coiled-coil domains, thereby influencing cell migration and tissue morphology (Sandeep et al., 2023). In uterine leiomyomas, *NAV3* expression is significantly reduced, which may be associated with the abnormal activation of the GnRH receptor signaling pathway. Studies have shown that the activation of the GnRH receptor signaling pathway is closely related to the growth of uterine leiomyomas, and changes in *NAV3* expression may regulate this pathway through a feedback mechanism, thereby affecting tumor progression (Kakar et al., 2025). The role of *NAV3* in the GnRH receptor signaling pathway may indirectly influence the development of the vulva, especially in processes involving hormonal regulation.

*ESR1* (Estrogen Receptor 1) is a nuclear receptor widely present in the reproductive systems of various animals, primarily regulating gene expression by binding to estrogen, and participating in the development of reproductive organs, gonadal differentiation, and the synthesis and secretion of sex hormones (Klinge, 2000). *ESR1* plays a key role in estrogen signaling, modulating the transcriptional activity of target genes by binding to estrogen response elements and other transcription factor binding sites, such as AP1 and CREB, thereby influencing the physiological functions of the reproductive system. In the reproductive systems of animals, the role of *ESR1* is particularly prominent (Revankar et al., 2005). Studies have shown that *ESR1* plays a decisive role in gonadal differentiation (Kohno et al., 2015; Tohyama et al., 2017). For example, in birds and reptiles, *ESR1* regulates gonadal sex differentiation by modulating the synthesis and signaling of estrogen (Ge et al., 2012). In the Chinese soft-shelled turtle (Pelodiscus sinensis), estrogen signaling mediated by *ESR1* can induce sex reversal in genetically male individuals, leading to the development of typical ovarian structures in females. This sex reversal is characterized not only by the formation of ovaries but also by the downregulation of male marker genes (such as *SOX9*, *DMRT1*, and *AMH*) and the upregulation of ovarian development regulators (such as *FOXL2*). These changes indicate that *ESR1* plays a central role in estrogen-induced sex reversal (Li et al., 2022). The development and function of the vulva are regulated by various hormones and neural signals, and *ESR1*, as an important estrogen receptor, may influence the morphology and function of the vulva by modulating local estrogen levels and gene expression.

In summary, *VIP*, *ESR1*, and *NAV3* each play significant roles in the development and functional regulation of the reproductive system through their respective biological functions. They may influence the morphology and function of the vulva by modulating blood flow in reproductive organs, hormonal signaling, neural innervation, and intercellular signaling. The identification of these genes provides important clues for in-depth research into the genetic regulatory mechanisms underlying vulva traits in pigs and lays a foundation for future molecular breeding and reproductive physiology studies. Future research can further explore the specific mechanisms of action of these genes in vulva development, as well as their interactions with other genes and environmental factors, to provide stronger theoretical support for improving pig reproductive performance.

## Conclusion

In this study, we employed GWAS, multi-population meta-analysis, and Bayesian fine mapping to identify several novel and pleiotropic QTLs associated with vulva traits in Large White pigs. Notably, the most significant SNP (rs3470833446), identified on chromosome 14 and associated with VW in PIC Large White pigs, explained 16.98% of the PVE. The most significant SNP (rs318318582), identified on chromosome 5 and associated with VAS in Canadian Large White pigs, explained 13.04% of the PVE. Additionally, a significant QTL influencing VAS on SSC5 (103.04 to 103.34 Mb) was identified in both the Topigs and Canadian Large White pig populations. A pleiotropic QTL on SSC4 (36.42 to 41.24 Mb) was found to simultaneously influence both VL and VW. The *VIP* and *ESR1* genes were identified as candidate genes for vulva width, while the *NAV3* gene was identified as a candidate gene for vulva angle score. These findings provide crucial insights into the genetic mechanisms underlying vulva traits and present valuable targets for MAS.

## Supplementary Material

skaf286_suppl_Supplementary_Material

## Data Availability

The phenotype data (https://doi.org/10.6084/m9.figshare.28581248) and SNP-chip data (https://doi.org/10.6084/m9.figshare.28582373) used in the present study are deposited in the figshare repository.

## Abbreviations

VL: vulva length
VW: vulva width
VAS: vulva angle scores
SNP: single-nucleotide polymorphism
PVE: phenotypic variation explained
GWAS: genome-wide associate analysis
iWGS: imputation to whole-genome sequence
LD: linkage disequilibrium
MAS: marker-assisted selection
QTL: quantitative trait locus
GS: genomic selection
