# Supplementary material for: Integrated genome-wide association studies, meta-analysis, and Bayesian fine mapping reveal novel quantitative trait loci’s and functional candidate genes for vulva traits in large white pigs
Source: J Anim Sci. 2025 Oct 14;103:skaf286. doi: 10.1093/jas/skaf286 (PMC12586332; doi:10.1093/jas/skaf286)

**Supplementary material**

**Integrated GWAS, Meta-Analysis, and Bayesian Fine Mapping Reveal Novel QTLs and Functional Candidate Genes for Vulva Traits in Large White Pigs**

Jin Zhou^*^, Xiaowen Qian^*^, Zijian Qiu^*^, Liming Xu^*^, Qian Liu^*^, Yanzhen Yin^*^, Jinfeng Ma^*^, Jianghui Yu^*^, David S. Casey^‡^, Lijing Zhong^§^, Qingbo Zhao^*^, Ruihua Huang^*,†, 1^, Pinghua Li^*,†, 2^

^*^ Key Laboratory in Nanjing for Evaluation and Utilization of Pigs Resources, Ministry of Agriculture and Rural Areas of China, Institute of Swine Science, Nanjing Agricultural University, Nanjing 210095, China

^†^ Huaian Academy, Nanjing Agricultural University, Huaian 223005, China

^‡^ PIC China, Shanghai 201107, China

^§^ Jiangsu Lihua food group Co.,Ltd, Changzhou 213000, China

^1^ Corresponding author: Ruihua Huang. Email: rhhuang@njau.edu.cn

^2^ Corresponding author: Pinghua Li. Email: lipinghua718@njau.edu.cn

Supplementary **Table S1** Descriptive statistics for VL, VW, and VAS.

| Population | Trait | N^1^ | Mean age ± SE | Min^2^ | Max3 | Mean^4^±SE^5^ | CV(%)^6^ |
| --- | --- | --- | --- | --- | --- | --- | --- |
| PIC | VL^7^ | 313 | 157.07±0.18 | 2 | 6 | 3.80±0.04 | 20.20 |
|  | VW^8^ | 313 |  | 2 | 5 | 3.08±0.03 | 16.06 |
| Topigs | VL | 1169 | 245.7±0.62 | 2.5 | 7 | 4.08±0.02 | 14.60 |
|  | VW | 1169 |  | 2.4 | 5 | 3.53±0.01 | 10.41 |
|  | VAS^9^ | 1169 |  | 1 | 5 | 3.80±0.03 | 22.64 |
| Canadian | VL | 715 | 188.64±0.32 | 2.7 | 8.4 | 4.88±0.04 | 19.77 |
|  | VW | 715 |  | 1.8 | 6.8 | 3.24±0.03 | 21.13 |
|  | VAS | 715 |  | 2 | 5 | 4.28±0.03 | 21.46 |

^1^Number of individuals with phenotypic records

^2^Minimum of phenotype

^3^Maximum of phenotype

^4^Mean of phenotype

^5^Standard error.

^6^Coefficient of variation

^7^Vulva length

^8^Vulva width

^9^Vulva angle score

Supplementary Table S2 GWAS results for vulva traits based on chip data.

| Population | Trait | Chr^1^ | N^2^ | Lead SNP | Position (bp) ^3^ | *P* value^4^ | Var (%)^5^ |
| --- | --- | --- | --- | --- | --- | --- | --- |
| PIC | VL | 5 | 1 | rs80907575 | 6599258 | 1.54E-05 | 7.48 |
|  |  | 6 | 1 | rs328327549 | 66624131 | 2.16E-05 | 7.96 |
| Topigs | VL | 5 | 10 | rs322368669 | 102362191 | 8.13E-08 | 9.74 |
|  |  | 6 | 1 | rs81476132 | 32528964 | 6.28E-06 | 7.14 |
|  |  | 8 | 14 | rs326031813 | 75668469 | 9.23E-07 | 7.01 |
|  |  | 14 | 1 | rs80965023 | 23991532 | 5.16E-06 | 2.27 |
|  | VW | 1 | 12 | rs81296558 | 14135085 | 1.87E-10 | 3.87 |
|  |  | 8 | 1 | rs81266478 | 3636759 | 1.35E-05 | 1.83 |
|  | VAS | 5 | 5 | rs322368669 | 102362191 | 1.03E-07 | 2.81 |
|  |  | 6 | 1 | rs81476132 | 32528964 | 1.26E-05 | 2.03 |
|  |  | 8 | 21 | rs326031813 | 75668469 | 1.05E-06 | 2.12 |
|  |  | 15 | 1 | rs81456495 | 136853482 | 1.80E-05 | 1.94 |
| Canadian | VL | 12 | 2 | rs80954325 | 8602233 | 2.16E-06 | 3.86 |
|  | VW | 2 | 2 | rs81362418 | 108541689 | 3.50E-06 | 3.63 |
|  |  | 4 | 2 | rs695915899 | 60111094 | 1.94E-05 | 3.24 |
|  | VAS | 1 | 1 | rs80837841 | 18614836 | 1.63E-05 | 3.26 |
|  |  | 5 | 1 | rs81318930 | 103209328 | 1.13E-05 | 2.97 |

^1^*Sus scrofa* chromosome, the same as below.

^2^The number of significant SNPs.

^3^Position of top SNP.

^4^*P* value according to the Wald test.

^5^Phenotypic variation explained by the top SNP.

**Supplementary Figure S1** The average consistency rate (a) and correlation (b) of imputation to whole-genome sequence by chromosome. The average accuracy of imputation shows in histogram before (red) and after (blue) filtering.


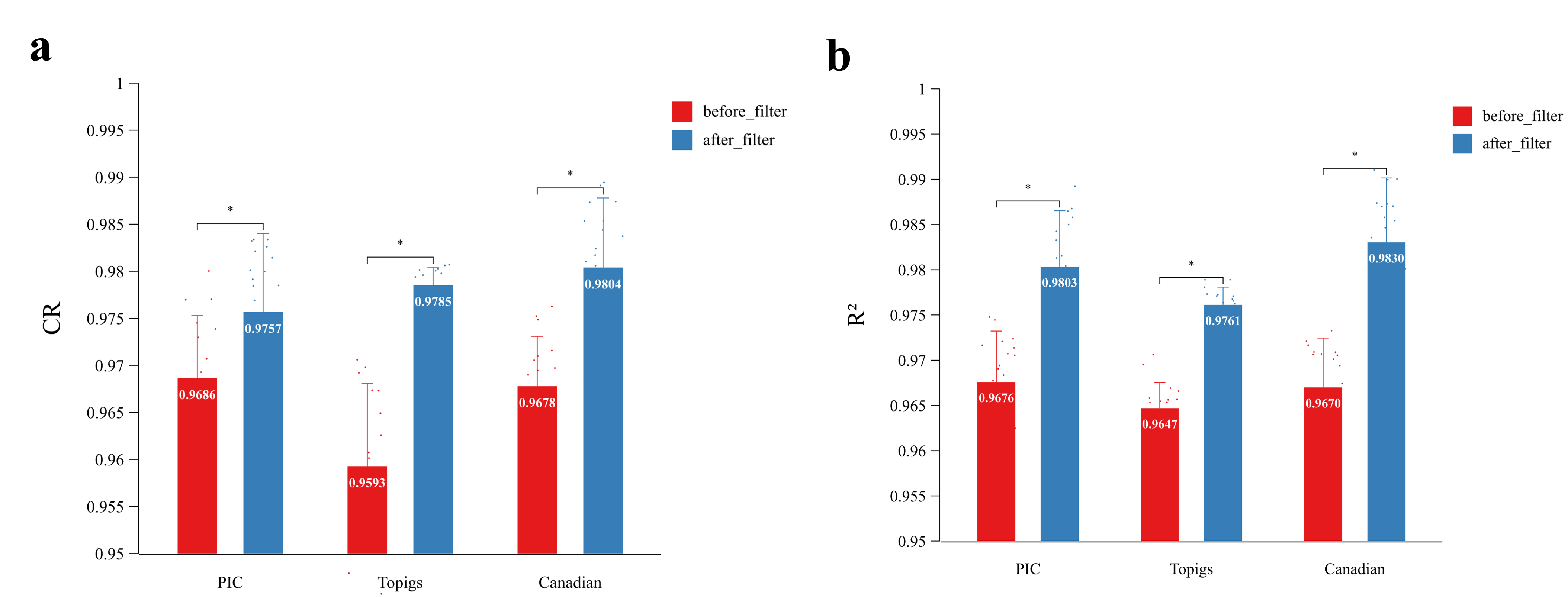


**Supplementary Figure S2.** Manhattan plot of GWAS based on chip data for vulva length (VL), vulva width (VW), and vulva angle score (VAS) traits. The results in (a) and (b) were PIC Large White pig, those in (c), (d) and (e) were Topigs Large White pig, while those in (f), (g) and (h) were Canadian Large White pig. Negative log10 P-values of SNPs (y-axis) were plotted against their corresponding genomic positions (x-axis). The horizontal solid and dashed lines represent the genome-wide significance and suggestive thresholds, respectively.


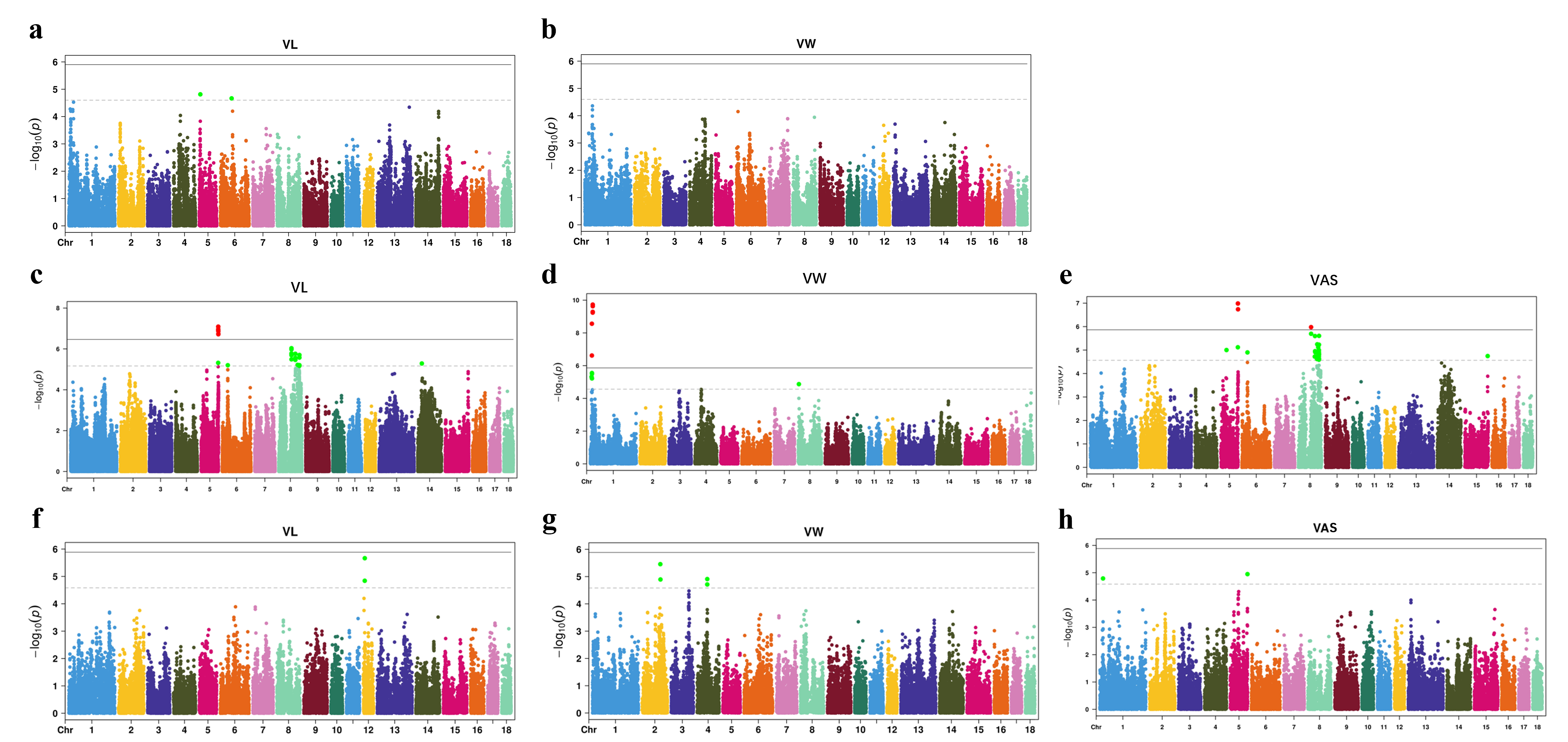


Supplementary Figure S3. The Q-Q plot of vulva traits in three strains of Large White pigs based on chip data GWAS.


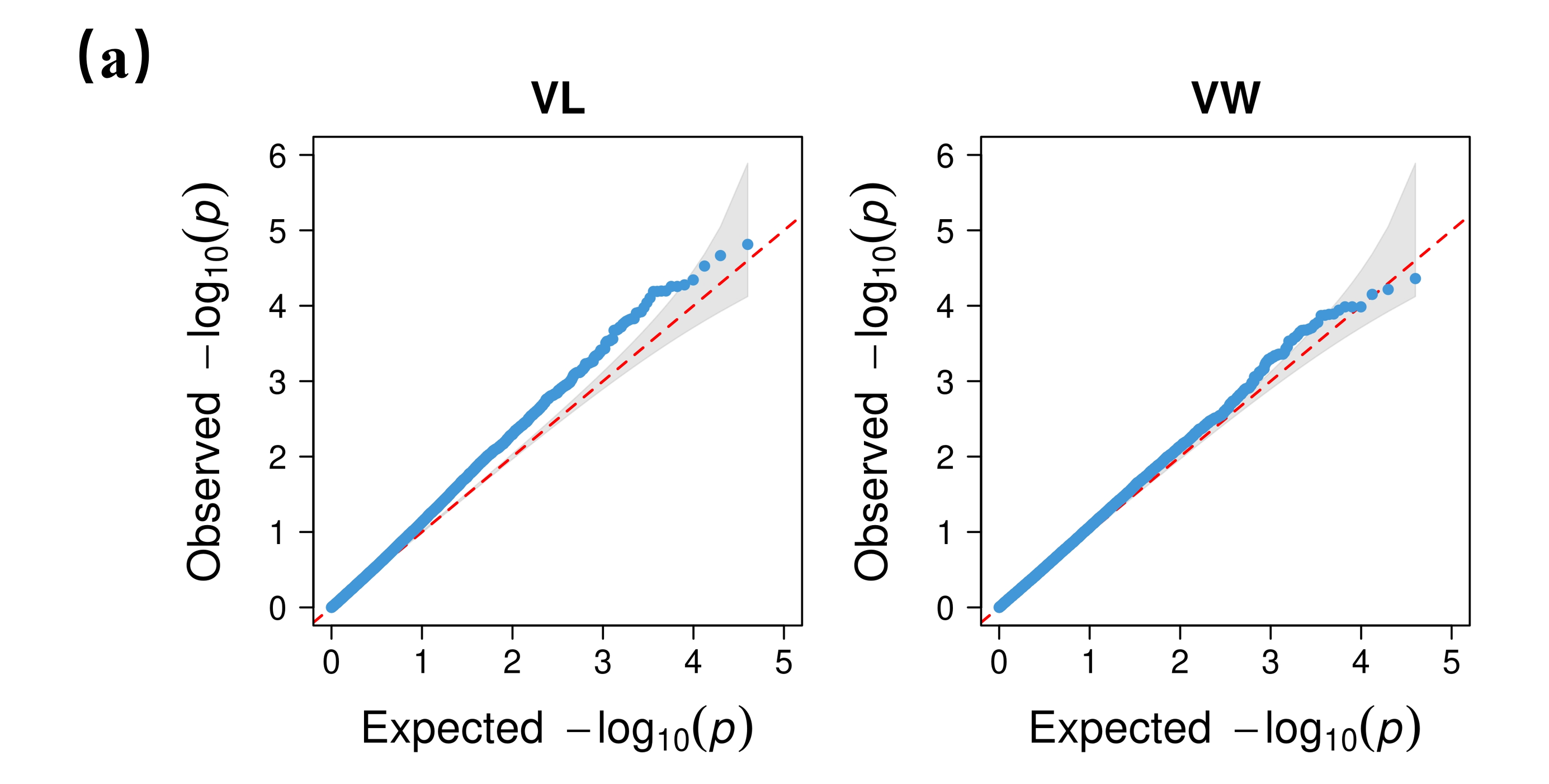


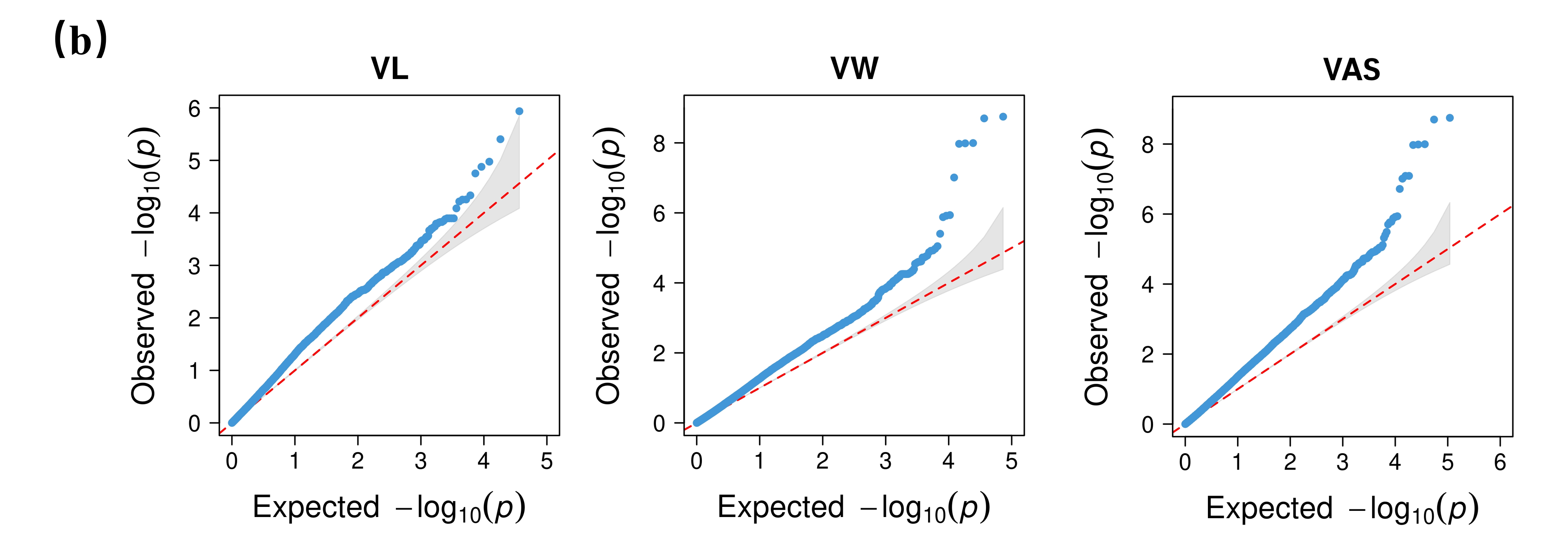


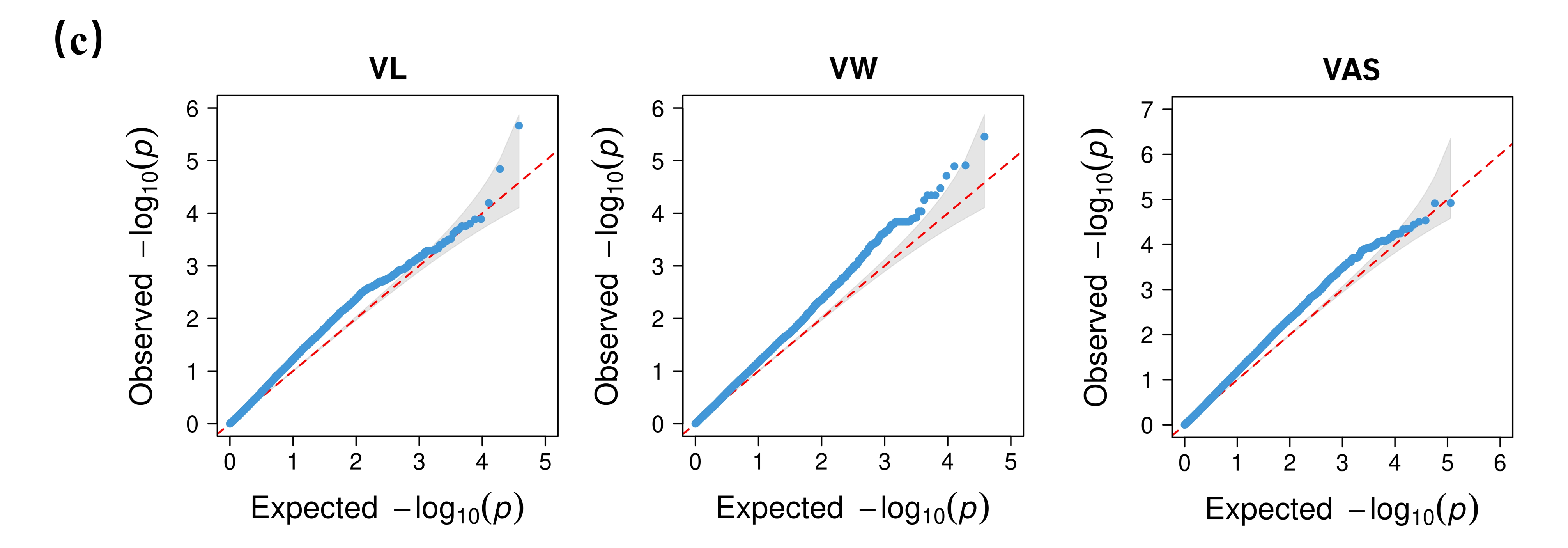


Abbreviations: From top to bottom are PIC (a), Topigs (b), and Canadian (c) Large White pigs.

**Supplementary Figure S4.**  The Q-Q plot of vulva traits in three strains of Large White pigs based on impute data GWAS.


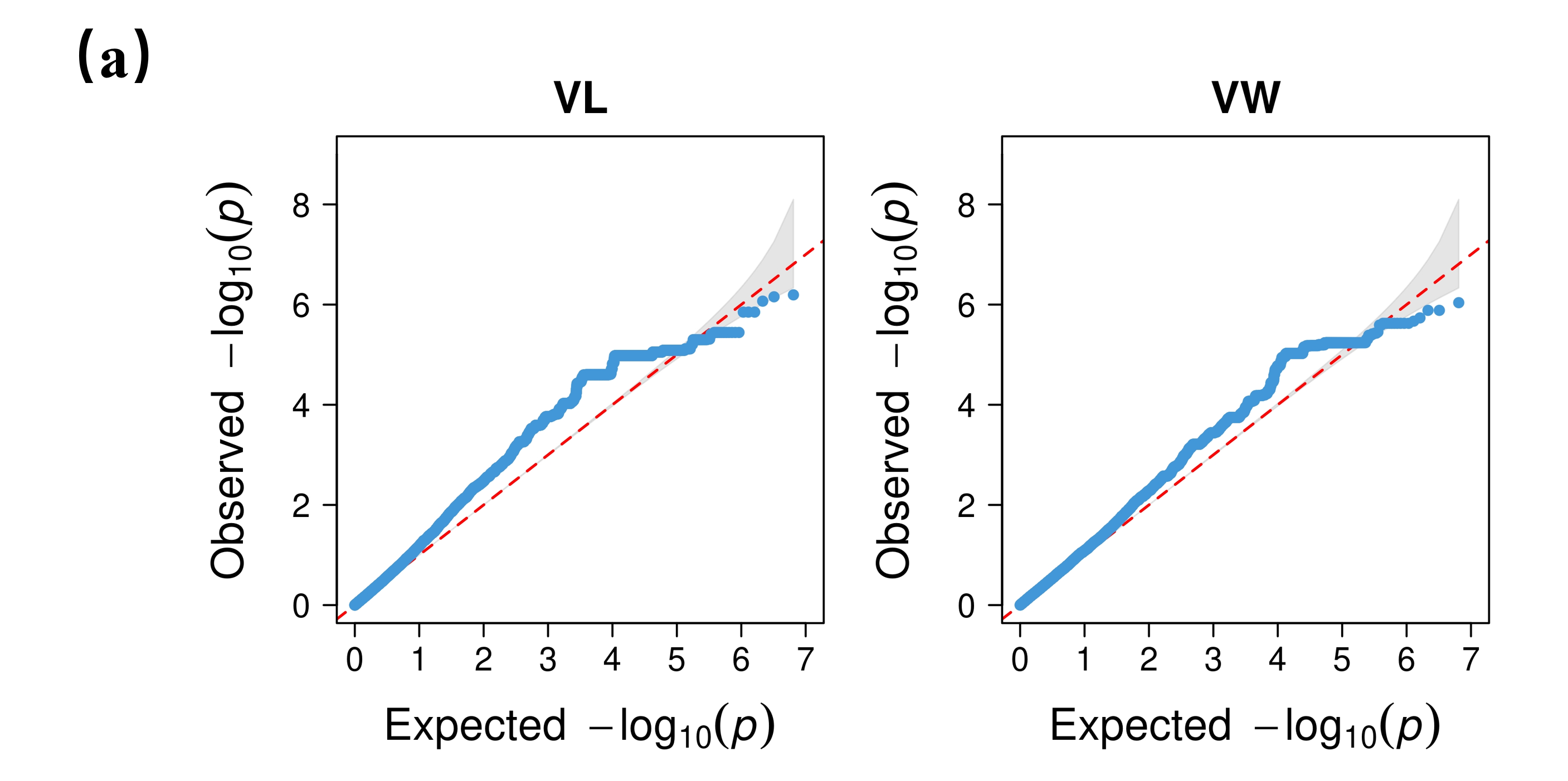


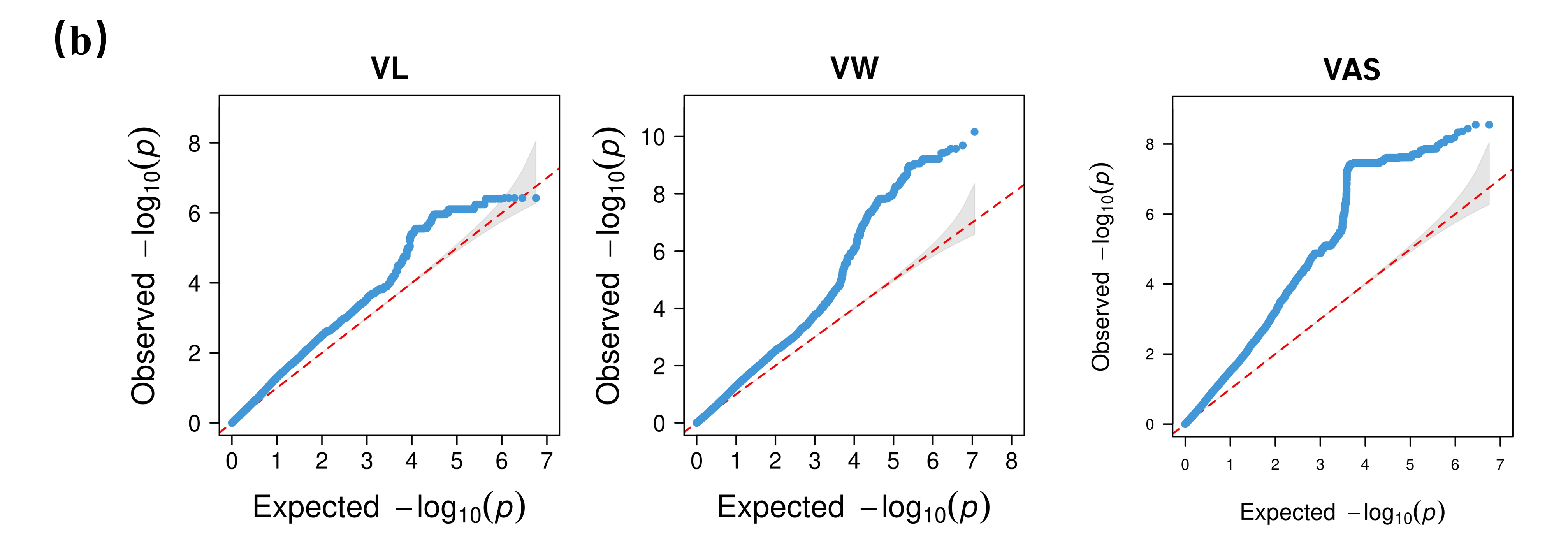


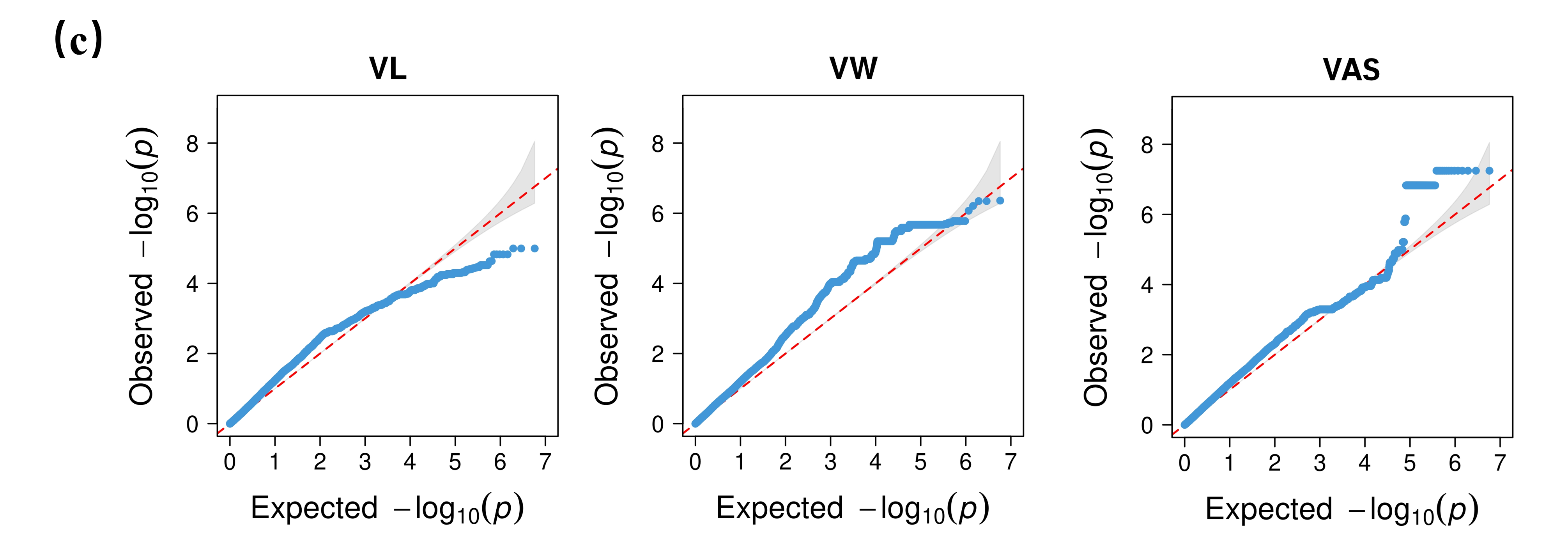


Abbreviations: From top to bottom are PIC (a), Topigs (b), and Canadian (c) Large White pigs.

**Supplementary Figure S5.**  The Q-Q plot of multi-population meta-analysis based on imputed
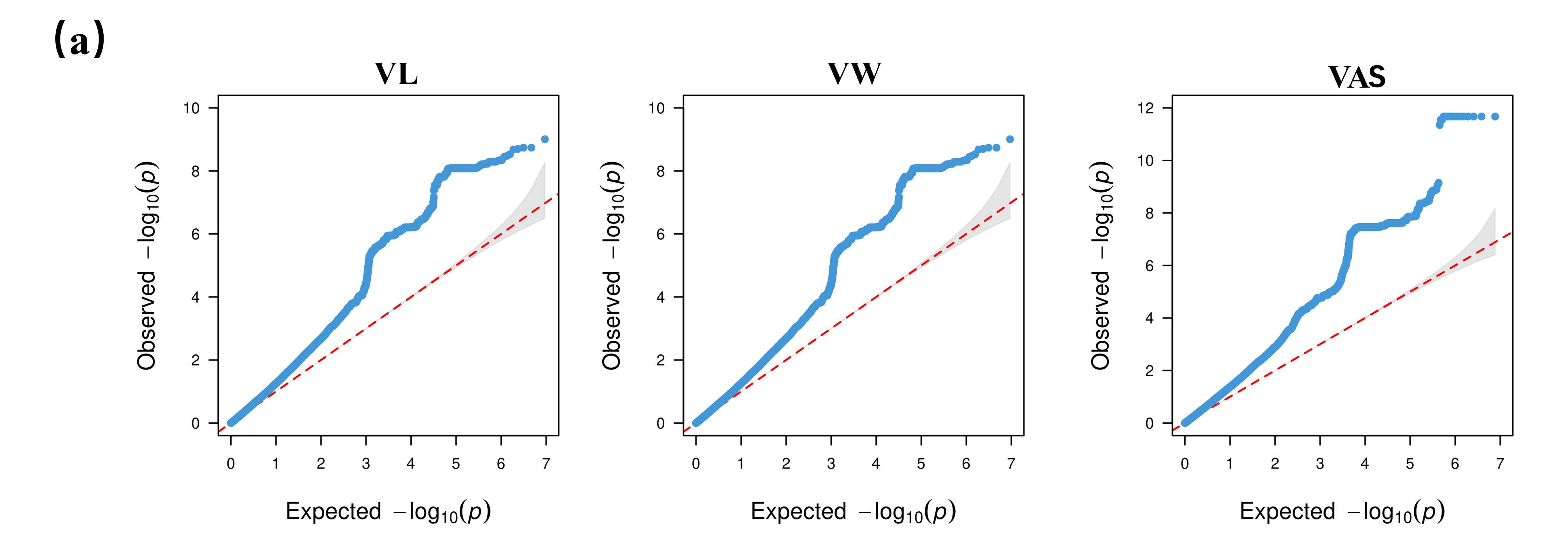

Supplement: skaf286_suppl_Supplementary_Material [file skaf286_suppl_supplementary_material.docx]
